# Hormonal regulation of *ethylene response factors* in tomato during storage and distribution

**DOI:** 10.3389/fpls.2023.1197776

**Published:** 2023-06-28

**Authors:** Me-Hea Park, Hae-Jo Yang, Siva Kumar Malka

**Affiliations:** Postharvest Research Division, National Institute of Horticultural and Herbal Science, Wanju, Republic of Korea

**Keywords:** ethylene response factors, hormonal regulation, storage temperature, fruit quality, ripening, ethylene, auxin, gibberellin

## Abstract

**Introduction:**

Ethylene response factors (ERFs) play a critical role in regulating hormone interactions that affect the shelf life of tomatoes. Understanding their regulation during storage and distribution can be highly beneficial.

**Methods:**

This study examined the effects of treatment with ethylene (ET), brassinosteroid (BR), auxin (AUX), and gibberellin (GA) on fruit ripening and the expression of 18 ripening-associated ERFs in tomato stored at 20°C (room temperature) for 10 d or 4°C (cold storage) for 14 d followed by 2 d at 20°C (retailer conditions).

**Results:**

The results showed that ripening was accelerated by ET and BR but was delayed by AUX and GA at room temperature. Cold storage delayed ripening in all groups, with ET and GA treatments showing the highest and lowest a* values, respectively. The effects of hormone treatment were consistent with room temperature when the fruits were transferred from cold storage to retail conditions. At room temperature, ERFs responsive to ET (ERF.B1, B2, B6, E2, and F1) and BR (ERF.E5, F2, and F3) were inhibited by AUX. ET-induced genes (ERF.C1, E1, F4, and H7) could be co-regulated by other hormones at cold storage. When the fruits were transferred from cold storage to retailer conditions, ERFs responsive to ET and BR were inhibited by GA. Additionally, ET-responsive ERFs could be inhibited by BR at room temperature, whereas ET could inhibit BR-responsive ERFs at retailer conditions. The same ERFs that were regulated by ET at room temperature were instead regulated by BR under retailer conditions, and vice versa.

**Discussion:**

These findings can help provide a better understanding of the complex hormone interactions regulating the postharvest physiology of tomato and in maintaining its quality and shelf life during storage and distribution.

## 1 Introduction

Tomato (*Solanum lycopersicum* L.) is one of the most widely consumed crops worldwide. However, its quality and shelf life is significantly affected by storage and distribution conditions (Jung et al., 2019). The interplay between phytohormones is crucial in regulating various physiological aspects of the fruit in response to different environmental factors (Kumar et al., 2014). Therefore, understanding the hormonal interactions that occur during tomato storage and distribution is essential to control and optimize the physiology and biochemistry of the fruit, ensuring its safety and quality.

Ethylene response factors (ERFs) are plant-specific transcription factors (TFs) that belong to the superfamily of Apetala 2/ethylene response factors (AP2/ERFs). They are characterized by the presence of the AP2/ERF DNA-binding domain. ERFs act downstream of the ET signaling pathway to mediate ethylene (ET) responses by regulating ET-responsive genes (Liu et al., 2016; Xie et al., 2016). The tomato genome contains 77 ERFs that are differentially expressed during ripening (Liu et al., 2016). Various aspects of fruit ripening, such as fruit color, softening, flavor, and aroma, are regulated by these TFs (Li et al., 2016; Li et al., 2019; Xie X. et al., 2016; Li et al., 2017; Tucker et al., 2017). ERFs are involved in a complex network of hormone cross-talk during fruit development and plant responses to environmental factors, which involves interactions between ET and other hormones such as auxin (AUX), gibberellin (GA), brassinosteroid (BR), and abscisic acid. For instance, *ERF.B3* and *D7* were responsive to both ET and AUX and integrate these two signaling pathways *via* regulation of AUX signaling components (Liu et al., 2014; Gambhir et al., 2022). Lorenzo et al. (2003) found that *ERF1* expression can be rapidly activated by either ET or jasmonate and that this activation can be synergistic when both hormones are present. ERF6 controls leaf growth under water-limiting conditions by fine-tuning the ET and GA/DELLA signaling pathways (Dubois et al., 2013). The *CaERF116* and *MsERF8* genes are induced by GA treatment (Chen et al., 2012; Deokar et al., 2015). As *ERFs* play an important role in integrating the signaling pathways of different hormones, understanding the regulation of *ERFs* by various hormones is essential to unravel the complex network of hormonal signaling pathways. However, the hormonal regulation of *ERFs* in tomatoes under different storage conditions is not well documented.

In this study, we characterized the regulation of *ERFs* by ET, BR, AUX, or GA in tomatoes stored at different conditions, including room temperature, cold storage, and retailer conditions.

## 2 Materials and methods

### 2.1 Cis-element analysis

The promoter sequences (2000-bp upstream of the translation start codon of *ERFs*) were obtained from the EnsemblPlants database (https://plants.ensembl.org/). All cis-acting elements were assessed by PlantCARE (https://bioinformatics.psb.ugent.be/webtools/plantcare/html/, accessed on 1 June 2022) (Lescot et al., 2002).

### 2.2 Plant materials and treatments

Cherry tomato (*S. lycopersicum* L. “Betatini”) fruits at mature-green stages were harvested during summer in Jungyeum, South Korea. Disease-free and intact fruits were sterilized with 2% sodium hypochlorite solution and washed with tap water twice. Following air drying at room temperature and removal of the pedicels, the fruits were divided into four groups and then treated with ET, BR, AUX, or GA. For ET and AUX treatments, the fruits were dipped in 1 mM ethephon solution (Inbio Corp., Jecheon, South Korea) and 0.45 mM 2,4-dichlorophenoxyacetic acid (Sigma-Aldrich, St. Louis, MO, USA), respectively, under a vacuum at 30 kPa for 5 min. For the BR treatment, the fruits were immersed in 6 μM brassinolide solution (Cayman Chemical Company, MI, USA) for 15 min. For the GA treatment, the fruits were dipped in a 0.5 mM GA3 solution (prepared in ethanol/distilled water [1:1000, v/v] containing 0.1% [v/v] Tween-20; Sigma-Aldrich) for 15 min. The fruit dipped with distilled water for 15 min was used as the control. Following the treatments, the fruits were kept in the dark at 20 ± 2°C (room temperature) with 90 ± 5% relativity humidity (RH) for 10 d or 4°C (cold storage) for 14 d followed by 2 d at 20 ± 2°C (retailer conditions).

### 2.3 Fruit color evaluation

Fifteen fruits were sampled per treatment to assess the fruit color. Skin color was monitored using a color difference meter (CR-400; Konica Minolta, Japan) and was reported based on Hunter’s scale: light (L*), red (a*), and yellow (b*).

### 2.4 Quantitative real-time polymerase chain reaction

qRT-PCR was performed on a CFX96 TouchTM Real-Time PCR detection system (Bio-Rad Laboratories, Hercules, CA, USA) as described by Park et al. (2018). The transcripts were amplified using the iQTM SYBR Green Supermix (Bio-Rad Laboratories) with specific primers (**Table S1**). qRT-PCR was performed under the following conditions: 95°C for 30 s, followed by 40 cycles of 95°C for 10 s and 55–58°C for 40 s. The relative gene expression level was calculated using the 2^-ΔΔCt^ method (Schmittgen and Livak, 2008) and normalized using the expression levels of the housekeeping gene *actin* (*solyc11g005330*). qRT-PCR analysis was performed using at least three biological replicates and two technical replicates.

### 2.5 Statistical analyses

Values are presented as the mean ± standard error. Samples were subjected to analysis of variance, and significant differences were determined using Duncan’s multiple range test. All analyses were conducted using SAS v.9.2 (SAS Institute, Cary, NC, USA). The *ERF* expression data and fruit color (a* values) were normalized, scaled, and used for the hierarchical clustering analysis and pattern correlation analysis (Pearson correlation coefficient) in the MetaboAnalyst 3.0 software (www.metaboanalyst.ca).

## 3 Results

### 3.1 Effect of hormone treatments on fruit ripening

**Figure 1:**
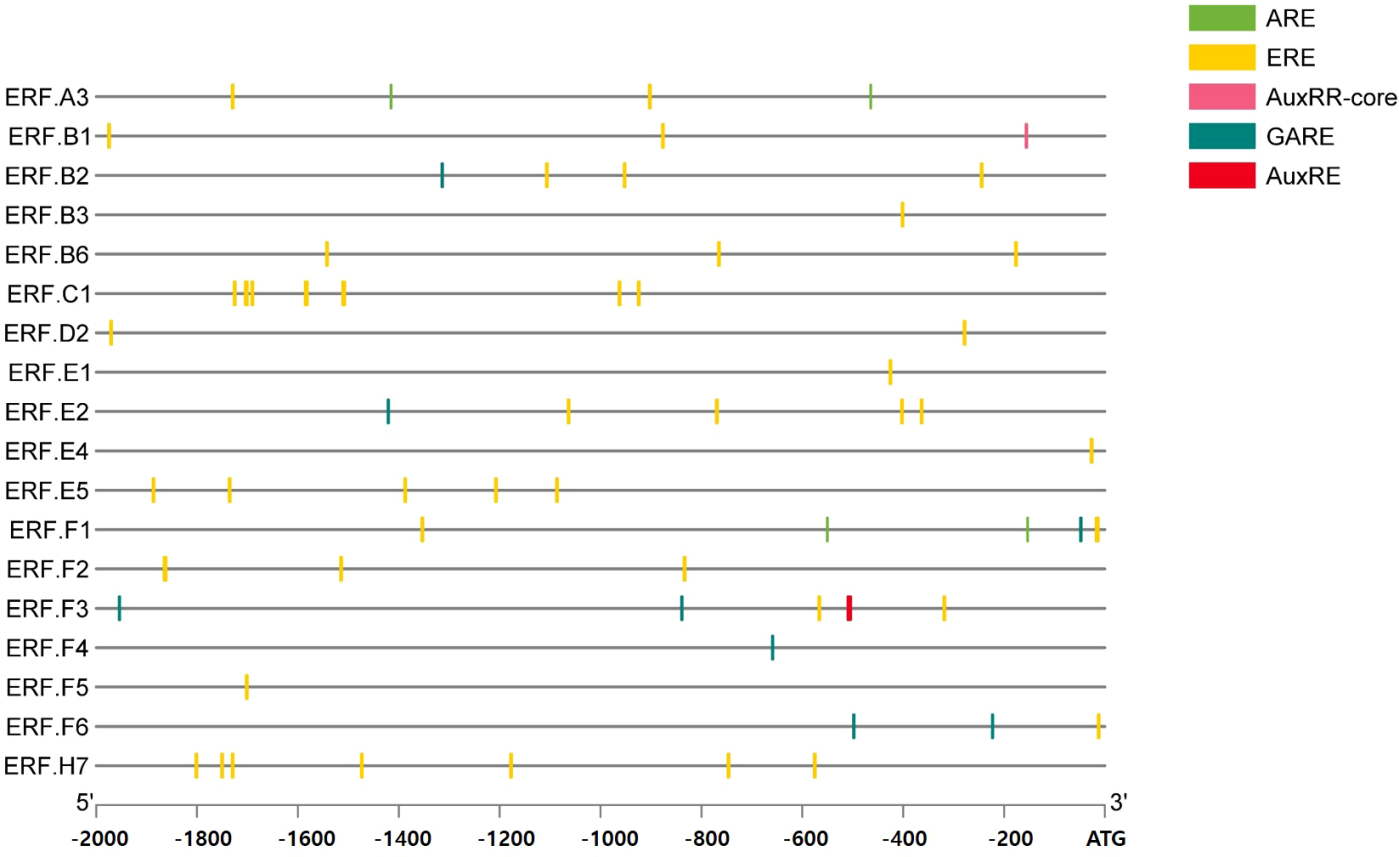
Promoter analysis for the presence of cis-regulatory elements. A total of 18 *ethylene response factors* (*ERF*s) were analyzed for the presence of cis-regulatory elements in the 2000 bp region upstream of the translation start codon. ARE, auxin response element; AUXRR-core, cis-acting regulatory element involved in auxin response; ERE, ethylene response element; GARE, gibberellin responsive element.

**Figure 2:**
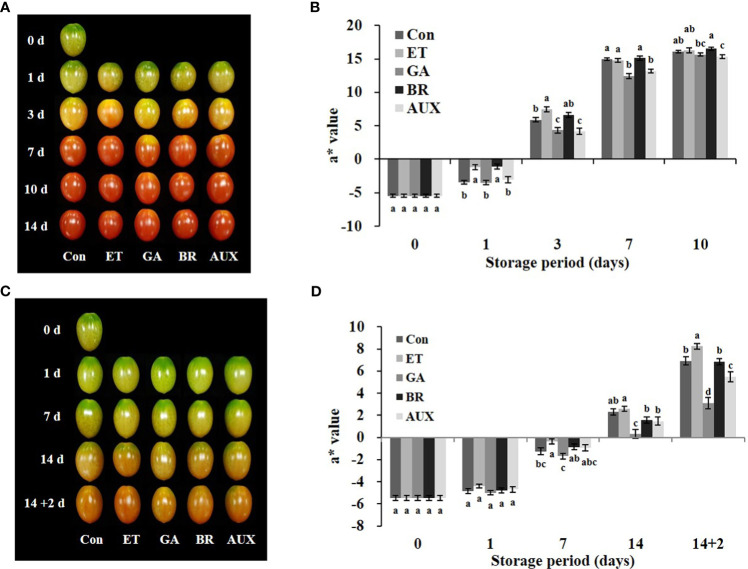
Effect of hormone treatments on tomato fruit ripening at different storage conditions. Changes in **(A)** color and **(B)** a* values in tomatoes stored at 20 ± 2°C (room temperature) for 10 d. Changes in **(C)** color and **(D)** a* values in tomatoes stored at 4°C (cold storage) for 14 d followed by 2 d at 20 ± 2°C (retailer conditions). Error bars represent standard error, and different letters on the graphs represent significant differences between the control and hormone treatments (*P* < 0.05). Con, control; ET, ethylene; AUX, auxin; BR, brassinosteroid; GA, gibberellin.

To explore the hormonal regulation of *ERFs*, we first analyzed the 2000 bp region upstream of the translation start codon of the 18 genes and identified the cis-elements related to ET, AUX, and GA (**Figure 1**). All the *ERFs* contained ET-related cis-elements. Additionally, all the genes contained cis-elements related to more than one hormone (**Figure 1**). Then, tomatoes at the mature-green stage were treated with ET, BR, AUX, or GA to determine the hormonal effect on ripening progression and the expression of *ERFs* during storage at room temperature for 10 d and/or cold storage for 14 d followed by 2 d at retailer conditions. The effect of hormones on fruit color was observed on day 3 at room temperature (**Figure 2A**). ET and BR treatments accelerated fruit reddening, with consistently higher a* (redness, Hunter scale) values than those of the control for 3 d at room temperature (**Figure 2B**). The AUX- and GA-treated fruits showed delayed color transition, as evidenced by consistently lower a* values than those of the control, ET-, and BR-treated fruits throughout storage at room temperature (**Figures 2A, B**). During cold storage, ripening was delayed in all treatment groups, and a visible color break was observed on day 14. ET-treated fruits showed the highest a* values, while GA-treated fruits showed the lowest values. BR- and AUX-treated fruits had values lower than those of the control fruits (**Figures 2C, D**). Under retailer conditions, the ripening process was accelerated in all treatment groups. However, ET- and GA-treated fruits still had the highest and lowest a* values, respectively. AUX-treated fruits had a* values in between those of control and GA-treated fruits, while BR-treated fruits did not significantly differ from control fruits (**Figures 2C, D**). Additionally, fruits treated with AUX and GA tended to have higher L* values than those of ET and BR-treated fruits on days 3–7 at room temperature and under retailer conditions (**Figures S1A, C**). However, there was no specific trend in the changes of the b* value between control and hormone-treated fruits at both storage conditions (**Figures S1B, D**).

### 3.2 Hormonal response of *ERFs* at room temperature

**Figure 3:**
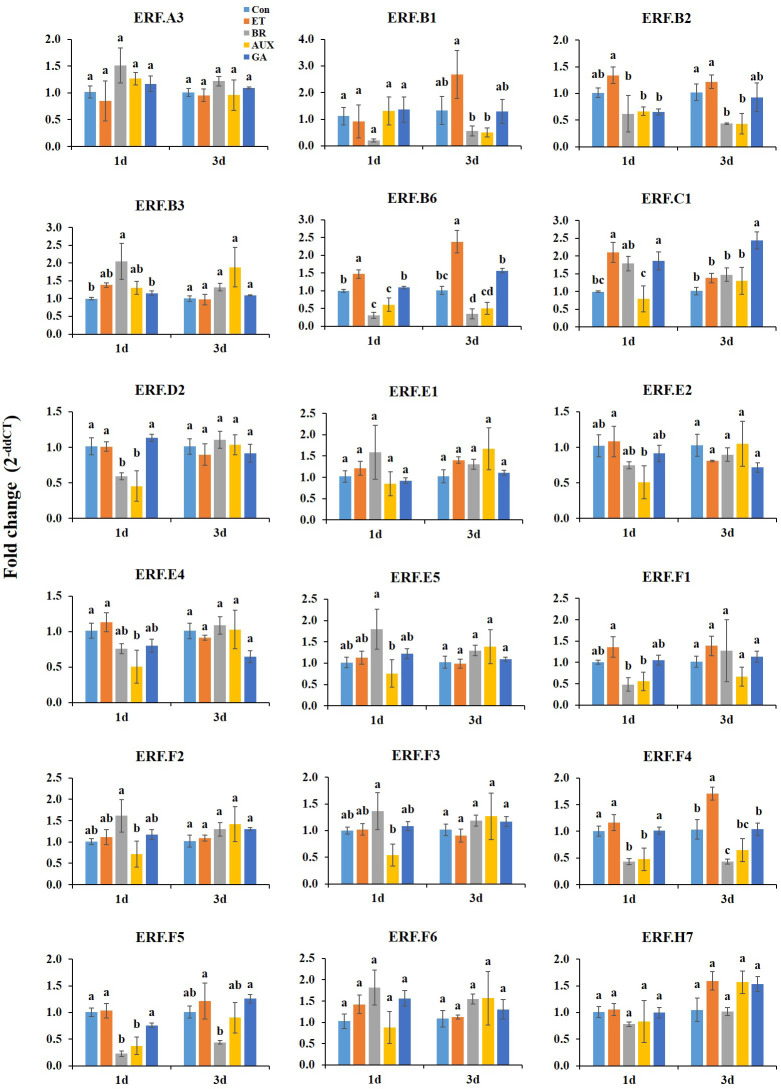
Effect of hormone treatments on the expression *of ERFs* in tomatoes during storage at room temperature (20 ± 2°C) for 10 d. Error bars represent standard error, and different letters on the graphs represent significant differences between the hormone treatments (*P* < 0.05). Con, control; ET, ethylene; AUX, auxin; BR, brassinosteroid; GA, gibberellin.

On day 1 at room temperature, ET treatment led to the expression of *ERF.B2*, *B6*, *E2*, and *F1*, while BR treatment increased the expression of *ERF.E5*, *F2*, and *F3*. However, AUX treatment reduced the expression of both ET- and BR-responsive *ERFs*, including *ERF.C1*, which was induced by all hormone treatments (**Figure 3**). BR was found to inhibit ET-responsive *ERFs*, except *ERF.E2*. GA treatment had no significant effect on most ET- and BR-responsive genes, except that it suppressed the ET-responsive *ERF.B2*. The expression of *ERF.B3* was enhanced by treatment with all hormones except AUX, and *ERF.F4*, *F5*, and *D2* were specifically reduced by AUX and BR treatments. Furthermore, *ERF.E4* was downregulated upon treatment with all hormones, except ET (**Figure 3**).

On day 3 at room temperature, ET treatment induced the expression of *ERF.B1*, *B6*, *F4*, and *F5* up to 2.5-fold, and these *ERFs* were downregulated by both AUX and BR treatments. ET-responsive *ERFs* were not affected by GA treatment, except *ERF.F5*, which was induced by GA treatment. Moreover, *ERF.C1* transcription was exclusively increased in GA-treated fruits. The level of *ERF.B2* was reduced in all fruits except those treated with ET. Hormone treatments had no significant effect on *ERF.B1* (day 1), *ERF.E2*, *F1*, *E5*, *F2*, *F3*, *B3*, *D2*, and *E4* (day 3), and *ERF.A3*, *E1*, *F6*, and *H7* (days 1 and 3) (**Figure 3**).

### 3.3 Hormonal response of *ERFs* in cold storage

**Figure 4:**
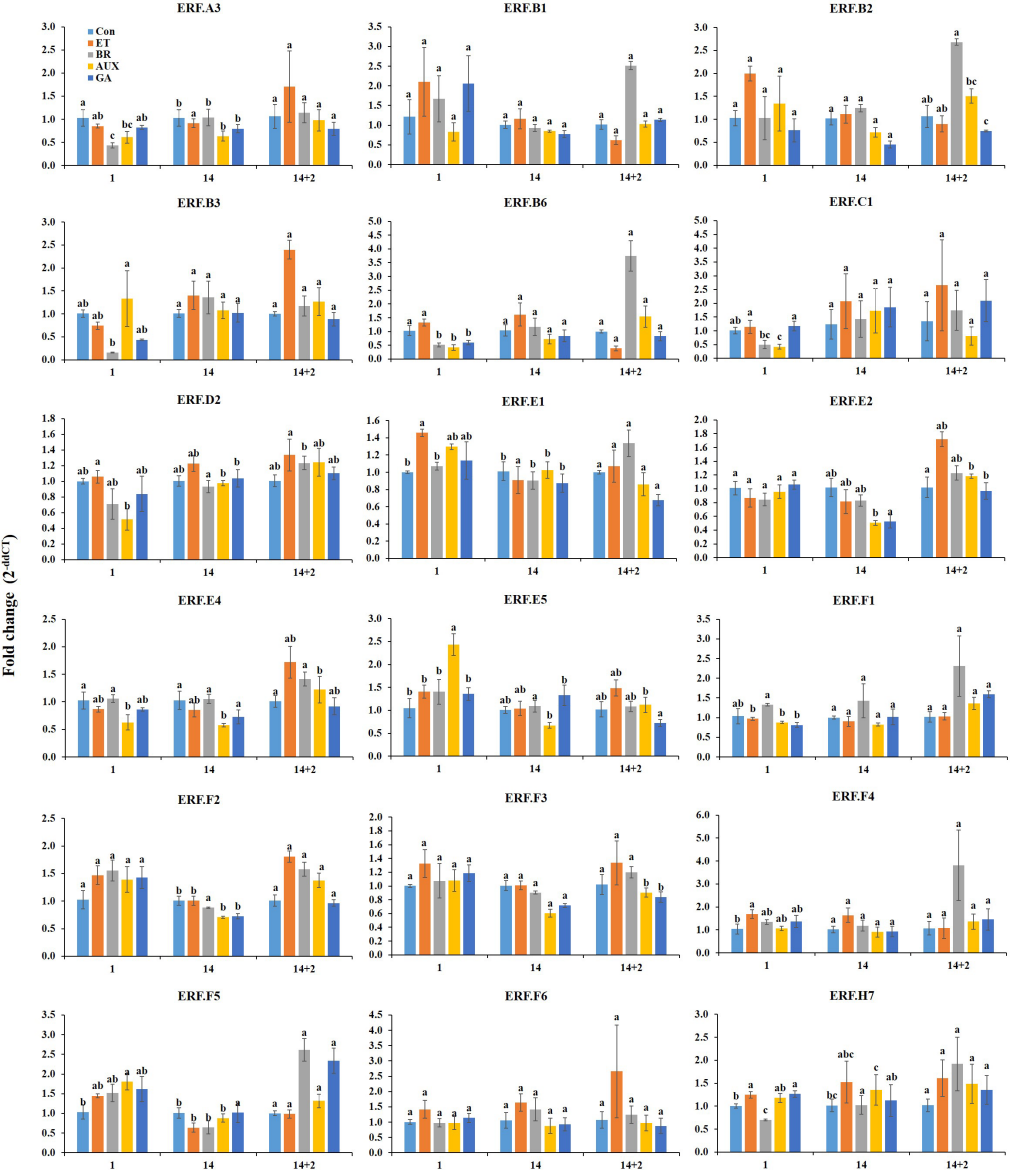
Effect of hormone treatments on the expression *of ERFs* in tomatoes during storage at 4°C (cold storage) for 14 d followed by 2 d at 20 ± 2°C (retailer conditions). Error bars represent standard error, and different letters on the graphs represent significant differences between the hormone treatments (*P* < 0.05). Con, control; ET, ethylene; AUX, auxin; BR, brassinosteroid; GA, gibberellin.

On the first day of cold storage, ET treatment increased the expression of *ERF. C1*, *D2*, *E1*, *F4*, and *H7*, while GA treatment also increased the expression of these genes except *ERF.D2*. AUX treatment decreased the expression of *ERF.D2* and *C1* and moderately increased the expression of *ERF.E1*, *F4*, and *H7*. BR treatment had varied effects, as it could trigger *ERF.F4* while decreasing *ERF.C1* and *H7*. The expression of *ERF.F1* was found to be most responsive to BR treatment but was reduced in other hormone treatment groups. *ERF.B3*, *E5*, and *F5* showed the highest level of responsiveness to AUX treatment, while *ERF.E5* was exclusively expressed in AUX-treated fruits. The transcript levels of *ERF.B3* and *F5* were considerably decreased in other hormone-treated fruits. Multiple hormones led to a decrease in *ERF.A3*, *B6*, and *E4*. However, on the first day of cold storage, the hormone treatments did not have a significant effect on the expression of *ERF.B1*, *B2*, *E2*, *F2*, *F3*, and *F6* (**Figure 4**).

On day 14 at cold storage, the hormone treatments had a significant impact on six *ERFs* (*ERF.B2*, *D2*, *E2*, *E4*, *E5*, and *F3*). *ERF.D2* and *B2* were most responsive to ET and BR treatments, respectively. BR treatment was found to suppress *ERF.D2*, while ET treatment had no effect on *ERF.B2*. In contrast, AUX and GA treatments could reduce the expression of *ERF.B2* but not *D2*. The response of *ERF.E4* and *E5* to AUX and GA treatments was the opposite, while ET and BR treatments had little effect on these genes. Additionally, *ERF.E2* and *F3* expression was found to be suppressed in AUX- and GA-treated fruits. *ERF. A3*, *B1*, *B3*, *B6*, *C1*, *E1*, *F4*, *F1*, *F2*, *F5*, *F6*, and *H7* were not significantly affected by hormone treatments after 14 d (**Figure 4**).

### 3.4 Hormonal response of *ERFs* at retailer conditions

Two days after transferring the fruits from cold storage to retailer conditions, twelve *ERF* genes were found to be significantly affected by hormone treatments. Interestingly, all these genes were most responsive to either ET or BR treatments. Specifically, *ERF.B3*, *E2*, *E4*, *E5*, and *F2* were highly responsive to ET treatment, with *ERF.B3* and *E2* were exclusively activated in ET-treated fruits. However, the activity of *ERF.E5* and *F2* could be reduced by GA treatment, while BR and AUX treatments showed moderate activation. BR treatment resulted in an increased expression of *ERF.B1*, *B2*, *B6*, *F1*, *F4*, *F5*, and *E1*. Notably, ET treatment was found to decrease the expression levels of some of the BR-responsive genes (*ERF.B1*, *B2*, and *B6*). AUX treatment had an inductive effect on some of the ET-responsive (*ERF.F2*) and BR-responsive (*ERF.B2*, *B6*, *F1*, and *F4*) genes, while its suppressive effect was limited to *ERF.E1*. Meanwhile, GA treatment was found to reduce the expression of ET-responsive (*ERF.E4* and *F2*) and BR-responsive genes (*ERF.B2* and *E1*). However, GA treatment increased the expression of the ET-responsive gene *ERF.E4* and BR-responsive genes *ERF.F1*, *F4*, and *F5*. The expression of *ERF.A3*, *F3*, *C1*, *D2*, *F6*, and *H7* were not significantly affected by treatment with any of the hormones (**Figure 4**).

### 3.5 Correlation analysis of *ERF* expression and fruit color

**Figure 5:**
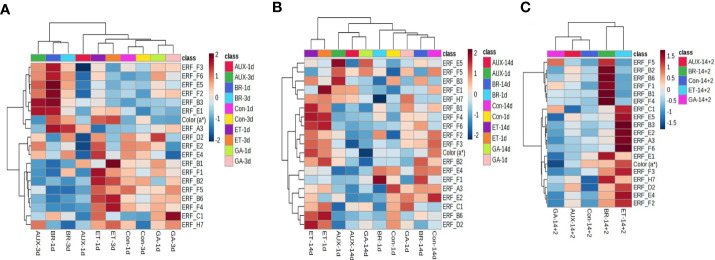
Correlation analysis of *ERFs* with fruit color in hormone-treated tomatoes at different storage temperatures. Heat map of hierarchical clustering analysis of fruits stored at **(A)** room temperature (20 ± 2°C) for 10 d and **(B)** 4°C (cold storage) for 14 d, **(C)** followed by 2 d at 20 ± 2°C (retailer conditions). Con, control; ET, ethylene; AUX, auxin; BR, brassinosteroid; GA, gibberellin.

To investigate the correlation between fruit color and *ERF* expression in hormone-treated tomatoes, hierarchical clustering and pattern correlation analysis were performed by combining the gene expression data with a* values. The heat map of hierarchical clustering analysis showed that the profiles of all treatment groups, except that of AUX-treated fruits, were closely correlated on days 1 and 3 at room temperature (**Figure 5A**). The a* values were grouped with *ERF.F3*, *F6*, *E5*, *F2*, *B3*, *E1*, and *A3*, and these genes, except *ERF.F6*, *E1*, and *A3*, were most responsive to BR treatment (**Figure 5A**). In cold-stored fruits, a distinct separation was observed between the profiles of ET treatment and those of other treatments. *ERF.B1*, *F4*, *F6*, *F2*, *F3*, and *B2* were clustered with a* values, and their profiles in ET-treated fruits were significantly different from those in fruits treated with other hormones (**Figure 5B**). *ERF.E5*, *F5*, *B3*, *E1*, and *H7* were closely clustered, and their expression was induced by AUX (**Figure 5B**). When fruits were transferred from cold storage to retailer conditions, a distinct separation was observed between the profiles of ET- and BR-treated fruits and other hormone-treated fruits (**Figure 5C**). The a* values were found to be correlated with several *ERFs*, including *ERF.E1*, *F3*, *H7*, *D2*, *E4*, and *F2*, which were most responsive to ET and/or BR (**Figure 5C**).

**Figure 6:**
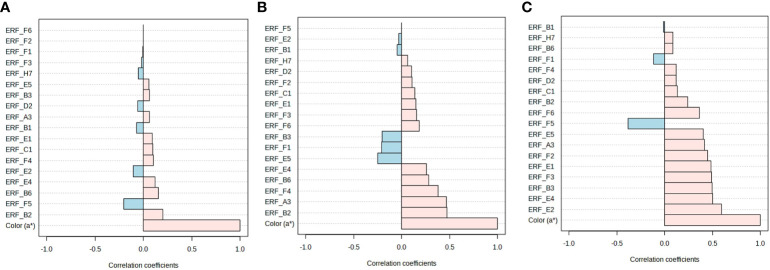
Pattern analysis of *ERFs* and fruit color in hormone-treated tomatoes at different storage temperatures. **(A)** Room temperature (20 ± 2°C) for 10 d. **(B)** 4°C (cold storage) for 14 d, **(C)** followed by 2 d at 20 ± 2°C (retailer conditions). Con, control; ET, ethylene; AUX, auxin; BR, brassinosteroid; GA, gibberellin.

Pattern analysis between a* values and gene expression revealed that certain *ERFs* (*ERF.B2*, *B6*, and *E4*) were positively associated with the a* values in fruits stored at room temperature, whereas other *ERFs* (*ERF.F5* and *E2*) were negatively associated (**Figure 6A**). When stored in cold temperatures, a* values were positively correlated with *ERF.A3*, *B2*, *B6*, *E4*, and *F4*, but negatively correlated with *ERF. B3*, *E5*, and *F1* (**Figure 6B**). Under retailer conditions, most *ERFs* were positively associated with color development, which suggests that ripening accelerated and quality changes occurred after cold storage regardless of hormone treatment (**Figure 6C**).

## 4 Discussion

In this study, we explored the hormonal regulation of *ERFs* and their impact on tomato fruit ripening during postharvest storage. Fruit ripening is a crucial process that affects the quality and shelf life of fruits. Our results showed that all *ERFs* contained ET-related cis-elements, indicating their potential regulation by ET (**Figure 1**). Furthermore, all *ERFs* contained cis-elements related to more than one hormone, indicating their potential regulation by multiple phytohormones (**Figure 1**). This finding is consistent with previous studies showing that plant hormones interact with each other to regulate plant growth and development (Wang and Irving, 2011).

### 4.1 Hormone treatments differentially affected tomato fruit ripening

The effect of ET, BR, AUX, and GA treatment on the ripening process and ERF expression was evaluated during storage at room temperature, cold temperature, and retailer conditions. The ripening process was characterized by measuring fruit color. At room temperature, the ripening process of the fruits was influenced by hormone treatment, being accelerated by ET and BR and delayed by AUX and GA. This was reflected in the fruit color as shown in **Figure 2A** and was consistent with previous research (Dostal and Leopold, 1967; Li J. et al., 2017; Malka and Park, 2022). While the a* values of control and ET- and BR-treated fruits were not significantly different after 7 d at room temperature, AUX- and GA-treated fruits consistently showed lower values throughout the storage period (**Figure 2B**). Of note, low storage temperatures inhibit fruit ripening (Vincent et al., 2020). Thus, during cold storage, the ripening process of fruits in all treatment groups was delayed (**Figure 2C**). This was evidenced by a delayed color break on day 14, with the highest and lowest a* values observed in ET- and GA-treated fruits, respectively (**Figure 2D**). When the fruits were transferred from cold storage to retailer conditions, the ripening process was accelerated in all treatment groups. Nonetheless, the effects of the hormone treatments remained largely consistent with those observed at room temperature (**Figures 2C, D**).

### 4.2 *ERFs* responsive ET and BR are inhibited by AUX at room temperature

The fruit quality of tomatoes rapidly changed at room temperature. ET is considered to be the primary regulator of fruit ripening, with BRs and AUX acting as modulators of ET signaling and biosynthesis (Kumar et al., 2014). In this study, ET treatment induced the expression of *ERF.B1*, *B2*, *B6*, *E2*, and *F1* in fruits stored at room temperature. Meanwhile, the transcript levels of *ERF.E5*, *F2*, and *F3* were increased by BR (**Figure 3**). Strikingly, the *ERFs* that were responsive to ET and BR treatment were suppressed in AUX-treated tomatoes concomitant with its inhibitory effect on ripening (**Figure 3**). The interaction between ET and AUX is well-established in various physiological processes of plant growth and development. In tomato fruit ripening, *ERF.B3* and *D7* play a critical role in integrating ET and AUX signaling (Liu et al., 2014; Gambhir et al., 2022). Furthermore, some of the ET-responsive *ERFs* were suppressed in BR-treated fruits (**Figure 3**), suggesting that they may be involved in a feedback regulation between ET and BR signaling. Additionally, ET-responsive *ERF.B2* and *B6*, along with AUX-suppressive *ERF.E4*, were positively correlated with color development (**Figure 6A**). *ERF.E4* has been shown to participate in ripening and carotenoid accumulation by integrating both ET-dependent and ET-independent regulatory activities, thereby enabling precise signal output modulation (Lee et al., 2012). These results suggest that the ET- and BR-responsive *ERFs* identified in this study may play crucial roles in the interplay between ET, BR, and AUX during the transition to ripening in tomato at normal temperature.

### 4.3 *ERFs* responsive to ET are co-regulated by BR, AUX, and GA under cold conditions

Low temperatures can significantly affect ET biosynthesis and signaling pathways in tomato fruit, which can result in delayed ripening (Kumar et al., 2014). During cold storage, the *ERFs* that were most responsive to ET and BR at room temperature remained largely unaffected by these hormones, potentially contributing to the observed ripening inhibition (**Figure 4**). While several *ERFs* responded specifically to ET during cold storage, they can also be co-regulated by other hormones, and the specific effect of each hormone may vary depending on the *ERF* (**Figure 4**). This suggests that these hormones may interact with the ET signaling pathway to regulate cold acclimation at low temperature.

Several studies highlighted the role of *ERFs* in the regulation of cold stress. For instance, *ERF105* has been found to be critical in freezing tolerance by operating in conjunction with CBF-regulon in *Arabidopsis* (Bolt et al., 2017). *ERF108* and *ERF9* from *Poncirus trifoliata* have been found to positively regulate cold tolerance by activating the raffinose biosynthesis gene and glutathione S-transferase gene, respectively (Khan et al., 2021; Zhang et al., 2022). Additionally, genes most responsive to BR (*ERF.B2*) and AUX (*ERF.B3* and *F5*) were reported to be involved in stress regulation (Tournier et al., 2003; Chen et al., 2008; Pan et al., 2012; Klay et al., 2014). Interestingly, the expression of AUX-responsive *ERF.B3* and *E5* were also negatively correlated with a* values (**Figure 6C**). These results suggest that these *ERFs* are involved in complex hormone interactions that govern cold response and ripening in cold storage.

### 4.4 *ERFs* responsive to ET and BR are inhibited by GA at retailer conditions

When fruits were transferred from cold storage to retailer conditions, the majority of these genes were activated by ET or BR (**Figure 4**). For example, *ERF.B3*, *E5*, and *F5* regulated by AUX on day 1 at cold storage were upregulated in ET- or BR-treated fruits at retailer conditions (**Figure 4**). *ERF.B3* responds to both ET and AUX, mediating salt and cold stress response in addition to its role in the regulation of ripening in tomato (Klay et al., 2014; Liu et al., 2014). Moreover, *ERF.E2* was found to be responsive to ET at both room temperature and retailer conditions but was downregulated by all the hormones during cold storage (**Figures 3**, **4**). *ERF.E2* integrates multiple signaling pathways that are responsive to biotic and abiotic stresses, including ET (Zhang et al., 2004). This suggests that the *ERFs* responsive to ET or BR have a dual function: cold response at low temperatures and ripening progression at retailer conditions. They may also be involved in the control of chilling injury. *ERF.E1*, which was most responsive to ET on day 1 during cold storage, was induced by BR at retailer conditions. *ERF.E1* has been found to be responsive to chilling injury in tomato (Bai et al., 2021).

Interestingly, certain *ERFs* most responsive to ET at room temperature were instead regulated by BR at retailer conditions; similarly, *ERFs* regulated by BR at room temperature were found to be most responsive to ET at retailer conditions (**Figures 3**, **4**). This indicates that although both room temperature and retailer conditions favored ripening, the hormonal regulation of *ERFs* was specific and depended on the storage conditions. However, *ERF.E2* was regulated by ET at both room temperature and retailer conditions (**Figures 3**, **4**). Furthermore, *ERF.F5* was negatively correlated with a* values at both room temperature and retailer conditions (**Figures 6A, C**), suggesting that these genes may play a significant role in the ripening and color development of tomatoes. Moreover, the inhibitory effect of AUX on ET- and BR-regulated *ERFs*, which was observed at room temperature, was largely absent under retailer conditions (**Figure 4**). However, GA had the ability to inhibit particular *ERFs* that were responsive to ET (*ERF.E5* and *F2*) and BR (*ERF.B2* and *E1*) (**Figure 4**). Furthermore, GA could co-regulate some ET- or BR-responsive genes at both room temperature and retailer conditions, suggesting complex interactions between these hormones in the harvested tomatoes.

**Figure 7:**
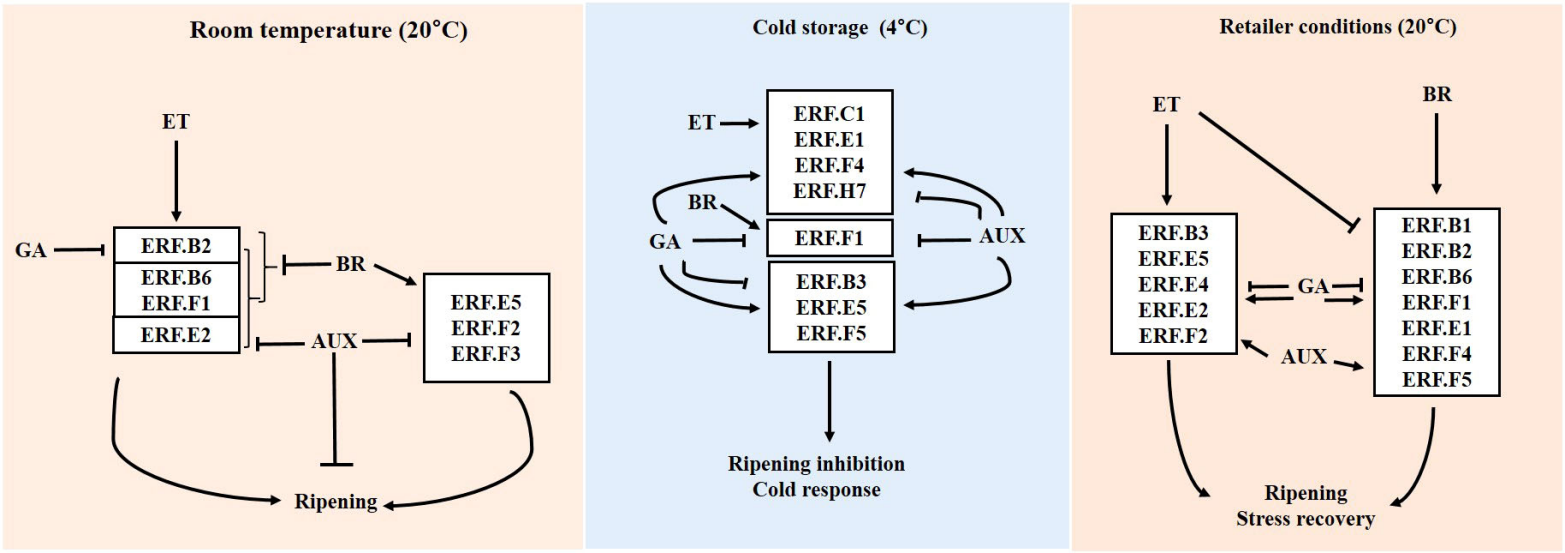
*ERF* regulation by hormones is affected by storage temperature. Solid and blunt arrows represent positive and negative regulation, respectively. Con, control; ET, ethylene; AUX, auxin; BR, brassinosteroid; GA, gibberellin.

Based on these results, we propose a model in which *ERFs* responsive to ET (*ERF.B2*, *B6*, *E2*, and *F1*) and BR (*ERF.E5*, *F2*, and *F3*) regulate fruit transition to ripening and its associated changes in harvested tomatoes at room temperature (**Figure 7**). *ERFs*, such as *B3*, *E1*, and *F5*, participate in intricate hormone interactions contributing to both cold acclimation and ripening inhibition in cold storage. Upon transfer of fruits from cold storage to retailer conditions, ET and BR may induce several *ERFs* to recover from chilling injuries and progress in ripening. AUX and GA can fine-tune the transition to ripening in tomatoes at room temperature and after being transferred from cold storage to retailer conditions, respectively (**Figure 7**).

### 4.5 Conclusion

In summary, we identified *ERFs* responsive to specific hormones at different storage temperatures. *ERFs* responsive to ET and BR were inhibited by AUX at room temperature and by GA at retailer conditions. Some *ERFs* may be involved in cold response at low temperatures and ripening progression and stress recovery after transfer from cold storage to retailer conditions. Overall, this study provides valuable insights into the complex hormonal regulation of *ERFs* associated with tomato postharvest ripening. These findings will be useful for further understanding dynamic hormonal interactions and for developing strategies to improve fruit quality and shelf life through regulation of hormone signaling pathways and *ERF* expression.

## Data availability statement

The original contributions presented in the study are included in the article/**Supplementary Material**. Further inquiries can be directed to the corresponding author.

## Author contributions

M-HP: conceptualization and supervision. H-JY: execution. SM: execution and writing–original draft preparation, and approved the submitted version. All authors contributed to the article and approved the submitted version.

## Supplementary material

Supplementary Table 1 List of primers.

Supplementary Figure 1 Effect of hormone treatments on changes in **(A)** L* and **(B)** b* values in tomatoes stored at 20 ± 2°C (room temperature) for 10 d. Changes in **(C)** L* and **(D)** b* values in tomatoes stored at 4°C (cold storage) for 14 d followed by 2 d at 20 ± 2°C (retailer conditions). Error bars represent standard error. Con, control; ET, ethylene; AUX, auxin; BR, brassinosteroid; GA, gibberellin.
