# Supplementary material for: Hormonal regulation of ethylene response factors in tomato during storage and distribution
Source: Front Plant Sci. 2023 Jun 28;14:1197776. doi: 10.3389/fpls.2023.1197776 (PMC10338070; doi:10.3389/fpls.2023.1197776)
Supplement: Supplementary Table 1 — List of primers. [file Table_1.docx]

| **Gene** | **Accession** | **Forward primer sequence (5` to 3`)** | **Reverse primer sequence (5` to 3`)** |
| --- | --- | --- | --- |
| ***SI-Actin*** | *Solyc11g005330* | TGTCCCTAT TTACGAGGGTTATGC | CAG TTAAATCACGACCAGCAAGAT |
| ***SI-ERF.A3*** | *Solyc05g052050* | TCGTCGGGAAACGGTTCCAT | GACATCCAACTTGCATGACACTTG |
| ***Sl-ERF.B1*** | *Solyc05g052040* | GAATGATGACGGAATTGTAATGAAGA | TTCCACAATCCCAAATTGAAGA |
| ***Sl-ERF.B2*** | *Solyc03g093560* | CCATCTCTGAACGTAGCAATACC | CATGGCCTCTGTCTAACTCC |
| ***Sl-ERF.B3*** | *Solyc05g052030* | CGGAGATAAGAGATCCAAGTCGAA | CTTAAACGCTGCACAATCATAAGC |
| ***Sl-ERF.B6*** | *Solyc01g090300* | GTGAAGAAGTGTAAGGAAGAG | GTGATAATGGAGGGAGGT |
| ***Sl-ERF.C1*** | *Solyc05g051200* | GCATTATCAATGAGGGGTCCTTG | TTAAAACAGCAGCTGGAGATAATCC |
| ***Sl-ERF.D2*** | *Solyc12g056590* | AGCTCAACCAACGTCGTTCCT | GGTTGTTGATCCGTAAAATCTCCTG |
| ***Sl-ERF.E1*** | *Solyc09g075420* | GTTCCTCTCAACCCCAAACG | TTCATCTGCTCACCACCTGTAGA |
| ***Sl-ERF.E2*** | *Solyc06g063070* | ACTTCGTGAGGAAACCCTGAAC | GTTACTAATATAAGTCATGTTGGGCTGAA |
| ***Sl-ERF.E4*** | *Solyc01g065980* | AGGCCAAGGAAGAACAAGTACAGA | CCAAGCCAAACGCGTACAC |
| ***Sl-ERF.E5*** | *Solyc12g049560* | TGAACACTGAACACAAGCC | GGTACATCCATATTTCCTTCTGAG |
| ***Sl-ERF.F1*** | *Solyc10g006130* | ACGAGCTTTCTTCTTTTCTCTCTCTAAA | GAAACTCGATATCCTTCTGTAAAATCTTC |
| ***Sl-ERF.F2*** | *Solyc07g064890* | GACGATTGCCATAGCGACTGTG | CGGCTTTCTGAAAGAGGAAGAAG |
| ***Sl-ERF.F3*** | *Solyc07g049490* | AGTAGTAAGGTGACCCGGATGAAG | CACCGATCATCCACCACAGA |
| ***Sl-ERF.F4*** | *Solyc07g053740* | GAGCTAATGGCTGATTTTTGTATATAAGTTC | AAATGGTAGAAACAGCACGAGAAAG |
| ***Sl-ERF.F5*** | *Solyc10g009110* | TGGAGCGAAAGCGAAAACTAA | GTCTGACTCGGACTCCGATTG |
| ***Sl-ERF.F6*** | *Solyc12g005960* | CCGATGGGGGAAGGTCATAG | CTGATCATCATCGCCACCGC |
| ***Sl-ERF.H7*** | *Solyc06g066540* | TAATTCCAAGCCAAGAAAGTCC | TCATCTGTGGTGATTTCTGAC |

**Table S1.** Primer sequences for quantitative RT-PCR amplification.
