# Supplementary figures and images for: Hormonal regulation of ethylene response factors in tomato during storage and distribution
Source: Front Plant Sci. 2023 Jun 28;14:1197776. doi: 10.3389/fpls.2023.1197776 (PMC10338070; doi:10.3389/fpls.2023.1197776)

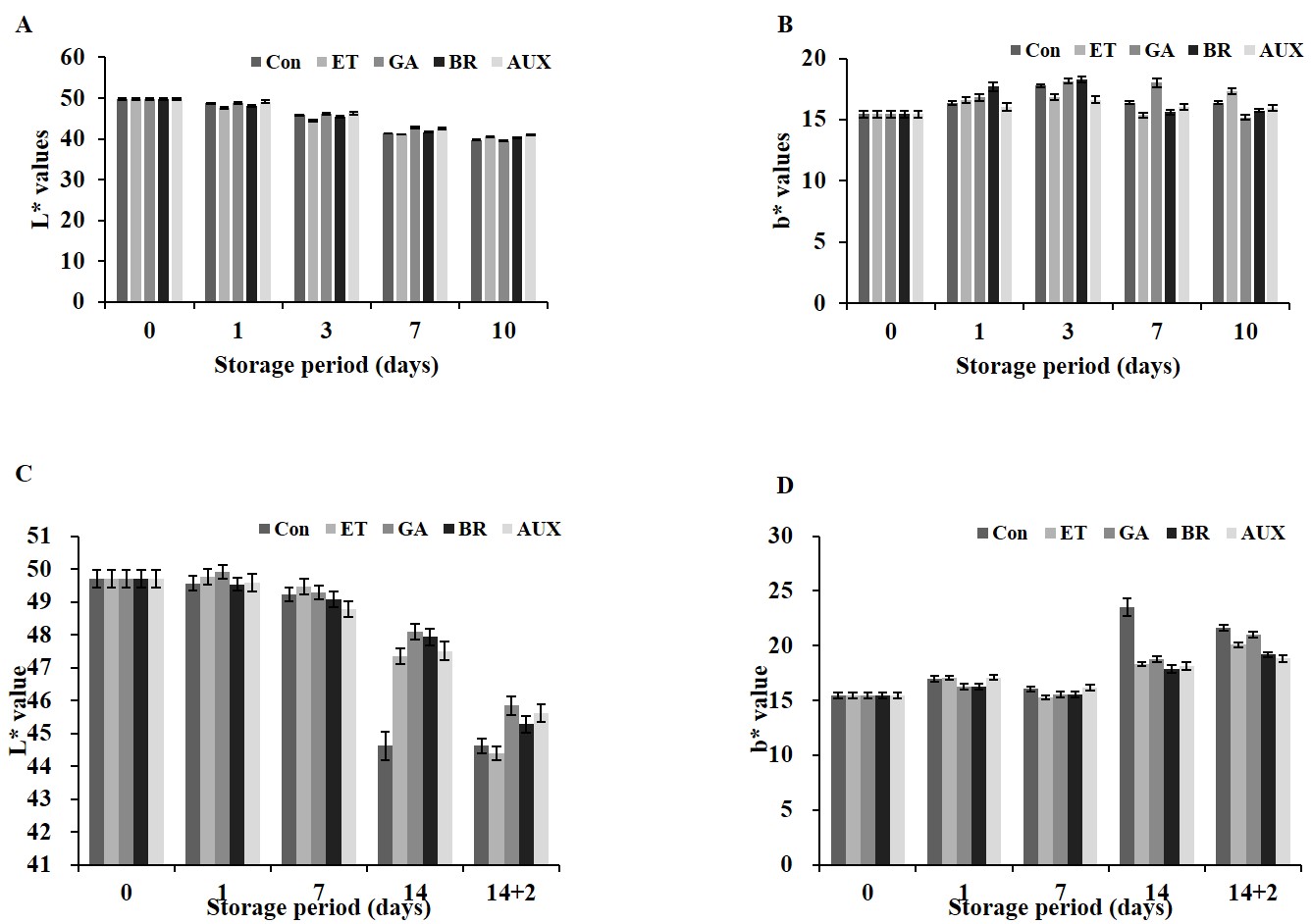

Supplement: Supplementary Figure 1 — Effect of hormone treatments on changes in (A) L* and (B) b* values in tomatoes stored at 20 ± 2°C (room temperature) for 10 d. Changes in (C) L* and (D) b* values in tomatoes stored at 4°C (cold storage) for 14 d followed by 2 d at 20 ± 2°C (retailer conditions). Error bars represent standard error. Con, control; ET, ethylene; AUX, auxin; BR, brassinosteroid; GA, gibberellin. [file Image_1.jpg]
